# Delayed Presentation of Gluteal Compartment Syndrome: The Argument for Fasciotomy

**DOI:** 10.1155/2016/9127070

**Published:** 2016-03-17

**Authors:** John E. Lawrence, Duncan J. Cundall-Curry, Kuldeep K. Stohr

**Affiliations:** Department of Trauma and Orthopaedic Surgery, Addenbrooke's Hospital, Cambridge University NHS Foundation Trust, Hills Road, Cambridge CB2 0QQ, UK

## Abstract

A male patient in his fifties presented to his local hospital with numbness and weakness of the right leg which left him unable to mobilise. He reported injecting heroin the previous morning. Following an initial diagnosis of acute limb ischaemia the patient was transferred to a tertiary centre where Computed Tomography Angiography was reported as normal. Detailed neurological examination revealed weakness in hip flexion and extension (1/5 on the Medical Research Council scale) with complete paralysis of muscle groups distal to this. Sensation to pinprick and light touch was globally reduced. Blood tests revealed acute kidney injury with raised creatinine kinase and the patient was treated for rhabdomyolysis. Orthopaedic referral was made the following day and a diagnosis of gluteal compartment syndrome (GCS) was made. Emergency fasciotomy was performed 56 hours after the onset of symptoms. There was immediate neurological improvement following decompression and the patient was rehabilitated with complete nerve recovery and function at eight-week follow-up. This is the first documented case of full functional recovery following a delayed presentation of GCS with sciatic nerve palsy. We discuss the arguments for and against fasciotomy in cases of compartment syndrome with significant delay in presentation or diagnosis.

## 1. Introduction

Compartment syndrome occurs due to increased pressure within a fascial compartment. Due to the fixed volume of these compartments, any increase in fluid within the interstitium will reduce the arteriovenous pressure gradient, ultimately resulting in reduced tissue perfusion and cell death [1]. Gluteal compartment syndrome (GCS) is a well-documented variant which most often occurs as a result of prolonged immobility [2–6], vascular injury [7], or iatrogenic injury [8, 9]. Though less common than compartment syndromes of the extremities, the symptoms are similar with the classical presentation involving a deep, burning pain out of proportion to any underlying injury. Paraesthesia and numbness are additional symptoms that are commonly part of the clinical picture. In addition, sciatic nerve dysfunction is commonly associated with GCS, despite the nerve being enclosed within a separate compartment. This is thought to be due to external compression on the arterial supply to the sciatic nerve, which most commonly arises from the medial circumflex femoral and inferior gluteal arteries [10–13].

Due to the large muscle mass within the gluteal compartment, patients with GCS are at increased risk of systemic complications. Of particular concern is rhabdomyolysis with profound myoglobinaemia, which results in acute renal failure that often warrants renal replacement therapy. Additionally, tissue necrosis often makes for difficult wound healing following fasciotomy [14].

We present a case of GCS presenting with profound sciatic nerve dysfunction and rhabdomyolysis with a long delay in diagnosis and treatment (over 56 hours). Despite this delay and systemic complications, the patient underwent emergency fasciotomy and made a strong postoperative recovery with a full return of function at eight weeks after discharge.

## 2. Case Report

**Figure 1:**
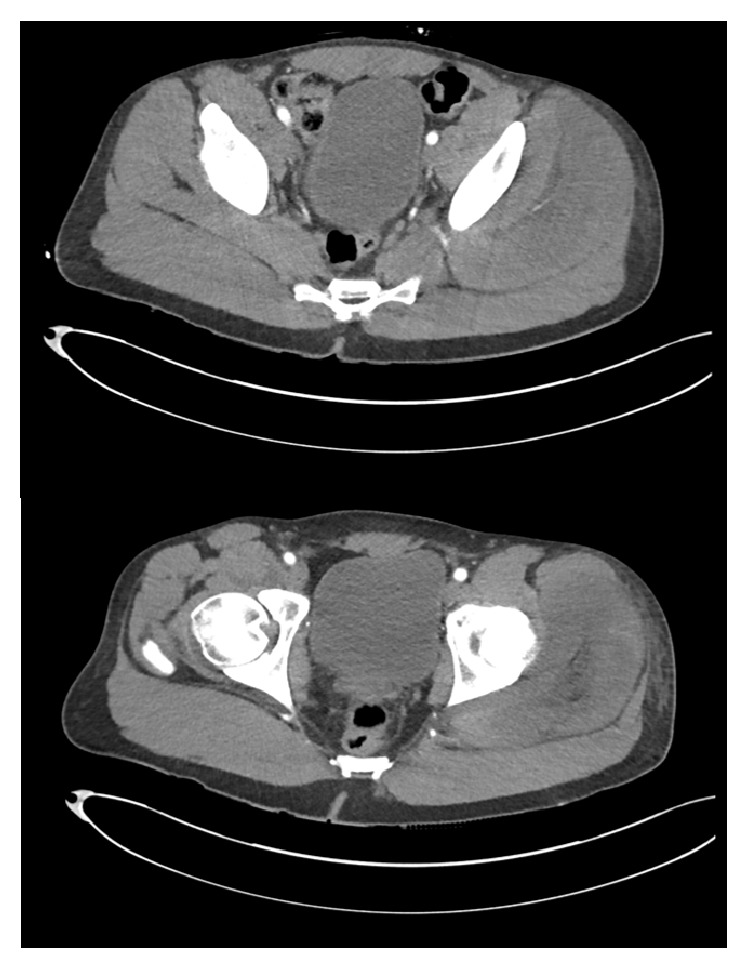
CTA of the gluteal vasculature showing intact vasculature with obvious swelling of the gluteal muscles within the left gluteal compartment, compared with the right.

A male patient in his fifties presented to his local hospital having awoken with numbness and weakness of the right leg which left him unable to mobilise. The patient was a known historic intravenous drug user who had maintained a period of abstinence over the preceding 12 months before relapsing and injecting heroin the previous morning. This was followed by a period of immobility, as he lay in a comatose state on the floor. On rousing he experienced pain in the gluteal region and an inability to walk, prompting him to seek medical attention. On arrival at the A&E department his condition was diagnosed as acute limb ischaemia secondary to intravenous drug-induced thromboembolism. The patient was commenced on treatment dose low-molecular weight heparin and transferred to a tertiary centre for an urgent Computed Tomography Angiogram (CTA, Figure 1). The angiogram showed patent vessels below the common iliac artery and was reported as normal. Detailed neurological examination revealed weakness in hip flexion and extension (1/5 on the Medical Research Council [MRC] scale) with complete paralysis in knee flexion and extension (0/5 on the MRC scale) and ankle plantar and dorsiflexion (0/5 on the MRC scale). Sensation to pinprick, vibration, and light touch was globally reduced and there was a loss of proprioception in the limb. Lower limb reflexes could not be elicited and the patient was in severe pain requiring regular high-dose oral morphine for analgesia. Blood tests revealed acute kidney injury with raised creatinine kinase, prompting treatment for rhabdomyolysis. In the context of these findings an orthopaedic referral was made with the suspicion of gluteal compartment syndrome. The patient was seen by the on-call orthopaedic team that confirmed the above examination findings and reviewed the CTA image with a musculoskeletal radiologist, who agreed that it showed significant swelling of the muscles in the gluteal compartment. The clinical picture together with the imaging findings was thought to be sufficient evidence for a diagnosis of gluteal compartment syndrome. Given the prolonged history not to measure compartment pressure was decided, as the results would not alter the management strategy and would be more likely to be normal than in an acute presentation, and taking measurements could cause a delay in treatment. The patient was subsequently taken to theatre for emergency fasciotomy, 56 hours following the onset of symptoms.

Fasciotomy was performed through a posterior approach with decompression of all gluteal muscles. The fascia lata was tense overlying an engorged, actively bleeding but contractile gluteus maximus, which was not debrided. Deep to this the gluteus medius contained some areas of friable, nonviable tissue which amounted to approximately half of the muscle. This tissue was progressively debrided down to contractile, bleeding tissue. Interestingly, the sciatic nerve was found to be intact with no obvious direct compression, other than slight impingement from piriformis which was released. The fasciotomy was left open and a negative pressure dressing was applied with the aid of nylon sutures. Examination on return to the ward revealed increased power in the limb with knee flexion and extension 3/5 MRC and ankle plantar and dorsiflexion exhibiting 4/5 MRC. Sensation gradually returned to the limb, becoming subjectively normal 36 hours after decompression, at which time the patient was requiring only oral nonsteroidal anti-inflammatory analgesia with oral morphine for breakthrough pain. A second and final debridement was performed 48 hours following the first procedure, with small areas of residual necrotic tissue removed from gluteus medius prior to washout with six litres of normal saline. A new negative pressure dressing was applied, prior to delayed closure 48 hours later.

**Figure 2:**
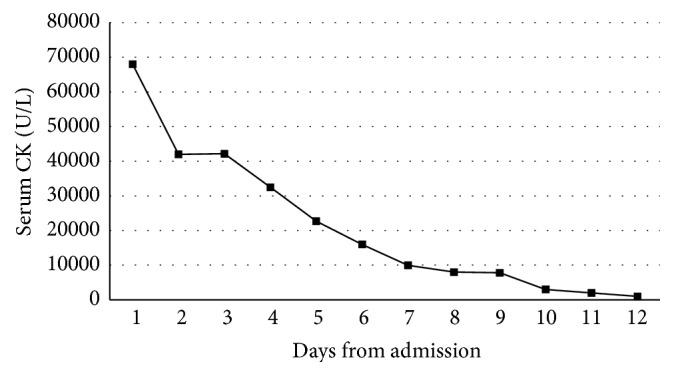
A graph showing total serum CK during admission.

The patient's recovery was complicated by worsening acute renal failure which resolved following haemofiltration (Figure 2). At discharge the patient was mobilising with a Trendelenburg gait due to loss of his hip abductors and 4/5 strength in all lower limb muscle groups. Pain was controlled by oral nonsteroidal anti-inflammatory medications. At eight-week follow-up, the patient had made a strong functional recovery with no residual sciatic nerve palsy. A full lower limb neurological examination revealed 5/5 MRC in all movements at all joints. He was mobilising unaided. Functionally, he was able to perform his activities of daily living unaided and reported full independence. Pain was not reported in the affected limb.

## 3. Discussion

Gluteal compartment syndrome is less common than compartment syndromes affecting the extremities, making delays in diagnosis and subsequent treatment commonplace [5, 15, 16]. Current guidelines for compartment syndrome recommend emergency fasciotomy within six hours, with necrosis of muscle and nerve tissue occurring beyond 5-6 hours [17]. Due to a delay in diagnosis, our patient did not receive fasciotomy until 56 hours after the onset of neurovascular compromise. This resulted in systemic complications (acute renal failure secondary to rhabdomyolysis) as commonly experienced by patients with GCS [1]. In addition, the initial misdiagnosis of acute limb ischaemia was treated with low-molecular weight heparin, which has been shown to worsen prognosis in compartment syndrome [18]. Despite this, the patient had made a full functional recovery at one month after discharge. To the best of our knowledge, this represents the longest recorded delay in treatment of GCS with a full functional recovery.

There is much debate surrounding the treatment of cases of compartment syndrome with delayed presentation. In such cases, tissue within the compartment will have become necrotic due to the lack of blood supply in the setting of sustained high compartment pressure. Several studies have highlighted the complications of surgical treatment in this setting. Finkelstein et al. described a series of nine delayed fasciotomies (defined as more than 35 hours after established compartment syndrome) in five patients in which one patient died from sepsis, and the remaining four required amputation due to localised infection and septicaemia [19]. In a retrospective analysis of victims of the Van earthquakes of 2011, Guner et al. found twelve of thirty-one patients that had fasciotomy required amputation [18]. In a similar but larger study, Zhang et al. retrospectively analysed treatment of Wenchuan earthquake victims and found sepsis to be significantly higher in patients treated with fasciotomy [20]. In addition, Reis and Better have described positive outcomes following conservative management of compartment syndrome in 35 patients and highlight various noninvasive techniques for lowering compartmental pressure including intravenous mannitol (in the absence of renal failure) and hyperbaric oxygen [21].

The argument for conservative management is further buttressed by cases in the literature that report the use of fasciotomy in delayed presentations of GCS with no subsequent improvement in symptoms [15, 16, 22]. Furthermore, there is published literature that does not support fasciotomy in cases where necrotic tissue is likely to be present within the compartment [23].

We have presented a case in which fasciotomy was performed over 56 hours after a compartment syndrome was established and where established necrosis of muscle within the compartment was found intraoperatively. Despite this, the patient's postoperative recovery was uncomplicated, and he gained immeasurable benefit by way of complete resolution of the sciatic nerve palsy with which he presented. Although intraoperative findings of an intact sciatic nerve were favourable and suggest eventual recovery without surgical intervention may have been possible, the speed with which the patient recovered after surgery strongly supports the choice of surgical decompression, which undoubtedly accelerated his recovery.

A further question raised by this case is whether the viability of the sciatic nerve despite raised gluteal compartment pressure is unusual. Had the nerve suffered irreversible damage secondary to ischaemia, the patient would not have recovered. To the best of our knowledge, there is no current literature on this topic, and anatomical studies analysing the effect of raised compartment pressure on the medial circumflex femoral and inferior gluteal arteries could shed light on whether sciatic nerve viability is likely to be the norm, rather than the exception.

In the setting of a tertiary referral centre, access to specialist wound care equipment, nursing staff trained in advanced wound care techniques, and an intensive treatment unit for the management of rhabdomyolysis minimised the likelihood of complications in this patient's recovery. Indeed, the main driving force behind the decision to take the patient to theatre was the knowledge that these facilities were in place, maximising the chances of an uncomplicated recovery and thus reducing the risks of surgery. As with any surgical patient, the decision to operate was also influenced by the gentleman's medical history. In this case, aside from the previous history of intravenous drug use, the patient was of good health with no chronic medical conditions. This was a further factor reducing the risks of surgery in this patient's case and clearly would not apply to patients of advanced age or with a complex medical history, who may be best managed conservatively.

Much of the literature concerning delayed fasciotomy relates to large-scale disasters, such as the 2011 Van earthquake. Such scenarios place great strain on resources and are unlikely to permit conditions as optimal as those experienced by our patient. Given the vast potential benefits for the patient, it is our opinion that there remains a place for fasciotomy in the treatment of delayed presentations of GCS in cases where optimal facilities and conditions exist.
